# Coronin 1C inhibits melanoma metastasis through regulation of MT1-MMP-containing extracellular vesicle secretion

**DOI:** 10.1038/s41598-020-67465-w

**Published:** 2020-07-20

**Authors:** Alicia C. Tagliatela, Stephanie C. Hempstead, Priya S. Hibshman, Max A. Hockenberry, Hailey E. Brighton, Chad V. Pecot, James E. Bear

**Affiliations:** 1 Department of Cell Biology and Physiology, University of North Carolina at Chapel Hill, Chapel Hill, NC 27599 USA; 2 Lineberger Comprehensive Cancer Center, University of North Carolina at Chapel Hill, Chapel Hill, NC 27599 USA; 3 Division of Hematology and Oncology, University of North Carolina at Chapel Hill, Chapel Hill, NC 27599 USA

**Keywords:** Membrane trafficking, Cancer

## Abstract

Coronin 1C is overexpressed in multiple tumors, leading to the widely held view that this gene drives tumor progression, but this hypothesis has not been rigorously tested in melanoma. Here, we combined a conditional knockout of Coronin 1C with a genetically engineered mouse model of PTEN/BRAF-driven melanoma. Loss of Coronin 1C in this model increases both primary tumor growth rates and distant metastases. Coronin 1C-null cells isolated from this model are more invasive in vitro and produce more metastatic lesions in orthotopic transplants than Coronin 1C-reexpressing cells due to the shedding of extracellular vesicles (EVs) containing MT1-MMP. Interestingly, these vesicles contain melanosome markers suggesting a melanoma-specific mechanism of EV release, regulated by Coronin 1C, that contributes to the high rates of metastasis in melanoma.

## Introduction

Melanoma is the deadliest form of skin cancer with a high propensity for metastatic spread^1,2^. The most common genetic drivers of melanoma are BRAF-activating mutations such as V600E, often found in conjunction with loss of tumor suppressors such as PTEN^3–5^. If caught in its early stages, melanoma is treatable by surgical resection, but prognosis worsens significantly with the occurrence of in-transit, regional, or distant metastasis. Clinically, up to 54% of metastatic melanoma patients show tumor dissemination to the brain, 77% to the liver, and 85% to the lung^6^. The high rate of metastasis in melanoma is poorly understood, but may be linked to lineage-specific factors that impact vesicular trafficking, secretion, degradation, and overall cell migration^7,8^.

The extracellular matrix (ECM) is an important substrate for tumor cell migration, but it also acts as a physical barrier whose degradation by matrix metalloproteinases (MMPs) is thought to be a critical step in tumor dissemination^9–11^. MMPs are a family of both transmembrane (designated “membrane-type” or “MT”) and secreted catalytic enzymes capable of cleaving ECM proteins and other substrates. Membrane-type 1 matrix metalloproteinase (MT1-MMP) is a particularly important pro-invasive MMP across many cancer types ^12^ and whose expression closely correlates with invasion and metastasis^13–15^. MT1-MMP is also unique in its ability to both directly cleave a wide range of ECM proteins including collagen types I, II, and III, laminin 1 and 5, and fibronectin, as well as activate pro-MMP2^16^.

The trafficking of MT1-MMP to and from the plasma membrane (PM) and other membrane structures is surprisingly complex, but critical for the protein’s function during tumor invasion and metastasis. Phosphorylation of the cytoplasmic tail initiates internalization of MT1-MMP from the PM by both clathrin-mediated and caveolin-mediated routes^13^. Internalized vesicles traffic through the endolysosomal pathway, resulting in redirection to other areas of the PM or to the lysosome for degradation^17^. MT1-MMP recycling has been shown to involve various flotilins^18^ and SNARE proteins^19–22^, as well as a variety of Rab proteins including Rab5, Rab7^18,22^, Rab8^23,24^, and Rab27a^25^. Interestingly, MT1-MMP can also localize to secreted extracellular vesicles (EVs) and these EVs are capable of degrading matrix at sites distant from the cell^19,26^. The mechanism of MT1-MMP trafficking to EVs is not well understood, nor is the significance of this phenomenon for metastasis clear.

Coronins are highly conserved F-actin binding proteins that regulate actin dynamics through multiple mechanisms including ADF/cofilin-mediated turnover and Arp2/3 complex-mediated branching^27,28^. Coronin 1C is a member of the Type 1 coronin family that has been implicated in lamellipodial protrusion, cell migration and, most recently, vesicular trafficking^29^. In pancreatic β-cells, Coronin 1C regulates endocytosis of insulin granule membranes and subsequent retrograde transport^30^. Coronin 1C also plays a role in endosome fission to membrane contact sites on the endoplasmic reticulum^31^, and directly binds to the melanosome- and endosome-sorting protein Rab27a in pancreatic β-cells^32^. Unlike other coronin genes, Coronin 1C expression is highly dynamic and is regulated through transcriptional control, as well as through miRNAs^33–35^. Coronin 1C is upregulated in several cancers^36–40^, and its depletion by RNAi has been shown to inhibit proliferation, migration, and transwell invasion in lung-squamous cell carcinoma^34^ and triple-negative breast cancer cells^35^. However, its role in cancer progression has not been rigorously tested in animal models of metastatic cancer and has not been functionally tested in melanoma at all.

Animal models of cancer are important tools for studying tumor cell behavior in physiological contexts, and genetically engineered mouse (GEM) models allow for the study of tumor progression in the presence of an intact immune system. However, GEM models have been difficult to use to study metastasis due to lack of control over the site and timing of tumor initiation. Recently, we modified an existing GEM model of melanoma by adding a Cre-inducible TdTomato fluorescent reporter allele^41^. Using this model, we developed a precise tumor initiation protocol using localized trans-dermal application of 4-hydroxytamoxifen to activate CreER expressed only in endogenous melanocytes. The TdTomato allele in this model allows primary tumors to be tracked by intravital imaging and disseminated cells to be detected by fluorescence-based methods such as flow cytometry. By combining this model and tumor initiation protocol with a conditional knockout allele, we evaluated the role of Coronin 1C in melanoma progression and metastasis.

## Results

### Pten/Braf melanoma tumors lacking Coronin 1C grow faster and metastasize more frequently

**Figure 1:**
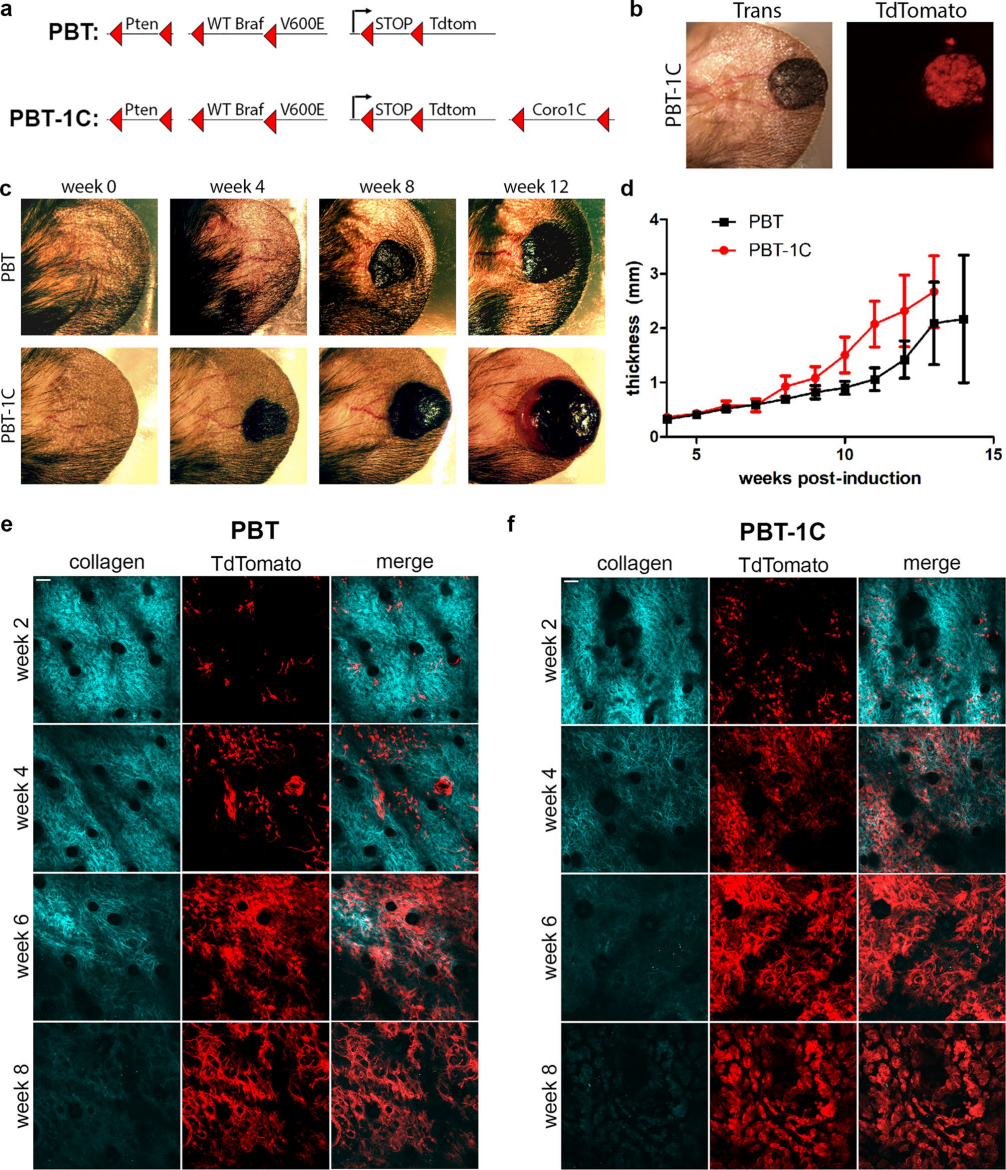
Coronin 1C-null melanoma grows faster than endogenous-expressing tumors.** (a) **Schematic diagram of two GEM models, PBT and PBT-1C. Upon 4-hydroxytamoxifen application, CreER expressed under the melanocyte-specific Tyrosinase promotor excises the DNA between LoxP sites, denoted with red triangles, resulting in Pten deletion, BRAF constitutive activation and TdTomato expression. In the PBT-1C model, Coro1C is also deleted. **(b)** Transillumination and red fluorescence for TdTomato imaging of a PBT-1C primary tumor 8 weeks after induction with 4-hydroxytamoxifen. **(c)** Transillumination of one PBT primary tumor and one PBT-1C primary tumor over a 12-week period following induction. **(d)** Quantification of the mean thickness + /− 95% CI of the ear and primary tumor (N of 15 for PBT and N of 16 for PBT-1C. **(e, f)** Representative multi-photon imaging of second harmonic signal of the bundled collagen layer and TdTomato expression in recombined melanocytes in the primary tumor of a PBT mouse ear **(e)** and a PBT-1C mouse ear **(f)** over an 8-week period post induction with 4-HT. Scale bars = 50 µm.

To test the role of Coronin 1C in melanoma growth and metastasis, we modified an existing GEM model by breeding in a Coronin 1C conditional knockout allele (Fig. 1a). The PBT model utilizes Cre/Lox recombination to delete the tumor suppressor gene Pten and constitutively activate the oncogene Braf in melanocytes^42^. The addition of a LoxP/stop/LoxP (LSL) TdTomato allele^43^ enables the tracking of induced tumor cells via fluorescent microscopy^41^ (Fig. 1b). The expanded model (designated PBT-1C) utilizes a previously validated conditional knockout allele of the Coro1C gene^44^, allowing us to directly compare tumor growth and metastases with and without Coronin 1C.

Primary tumors were induced on the ears of mice using a previously developed topical tamoxifen application protocol to activate CreER expressed in the mouse’s endogenous melanocytes using the Tyrosinase promoter. Primary PBT-1C tumors (lacking Coronin 1C) grew to a palpable mass four to five weeks after induction with tamoxifen, while it took approximately six to eight weeks for a palpable mass to form in the PBT animals (Fig. 1c). Over the course of 13 weeks, primary tumors lacking Coronin 1C grew deeper into the tissue significantly faster than their PBT counterparts (Fig. 1d). Multiphoton imaging of the TdTomato + tumor cells and the bundled collagen fibers (visualized via second harmonic imaging) within the ear revealed that the tumor cells increased in number faster when they lacked Coronin 1C, and that the collagen fibers degraded more quickly than the PBT control tumors (Fig. 1e, f). In addition, immunohistochemical staining of the primary tumors revealed stronger CD31 staining around blood vessels in PBT-1C primary tumors (Suppl. Fig. 1) suggesting increased angiogenesis^45–47^. Although there was a trend of larger blood vessels measured in PBT-1C tumors at 10 weeks post-induction compared to the control PBT counterparts, this change was not significant.

**Figure 2:**
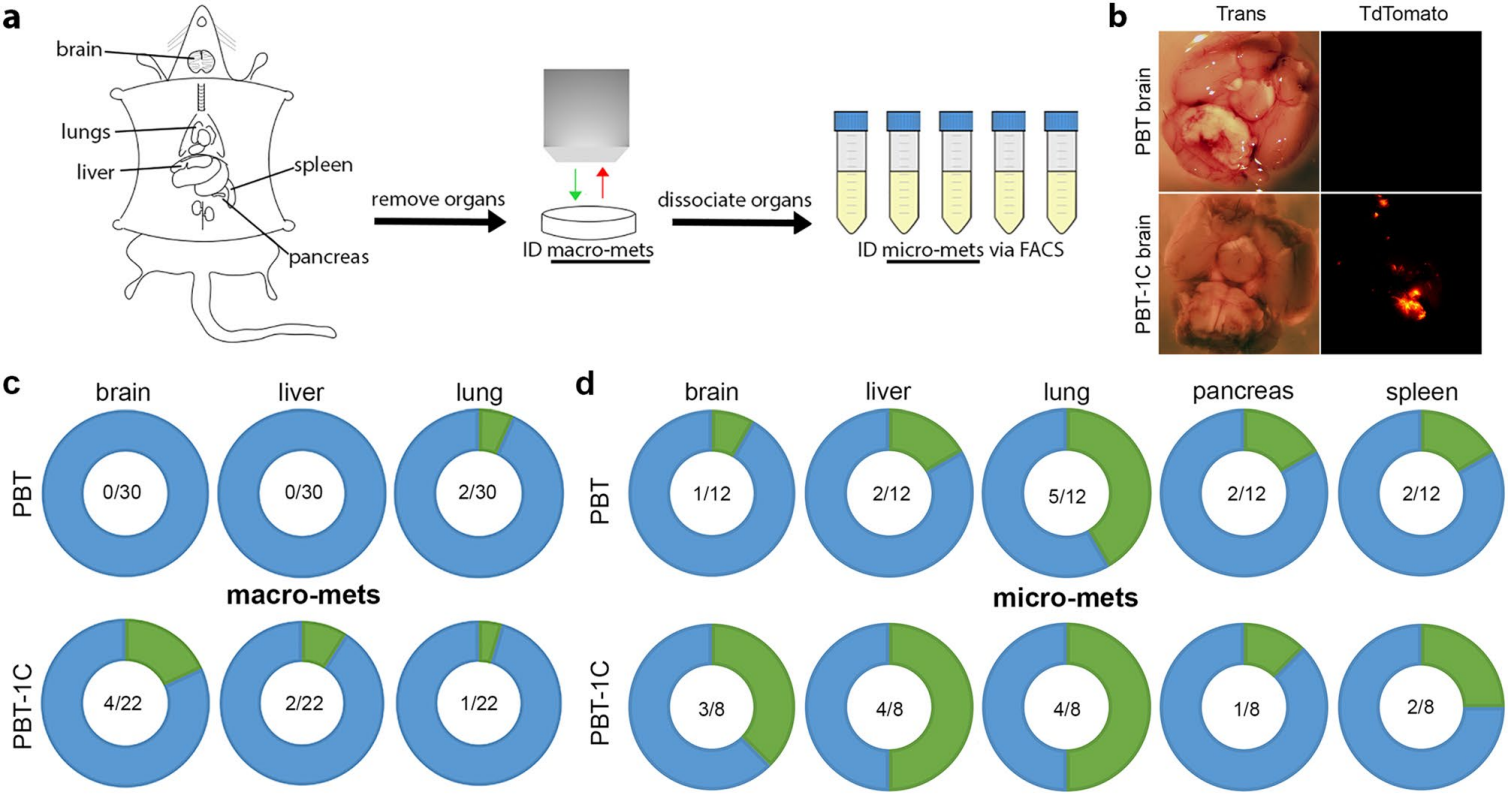
Loss of Coronin 1C in melanoma results in more distant metastases. **(a)** Schematic diagram of macro- and micro-metastasis identification in mouse models. Organs are removed and macro-metastases are identified with a stereo microscope via TdTomato signal. Organs are then dissociated to a single cell suspension and micro-metastases are identified by FACS sorting. **(b)** Example of a macro-metastasis identified in a PBT-1C mouse brain 13 weeks after induction. For comparison is a PBT brain with no visible metastases 19 weeks after induction. **(c)** Donut plots displaying the number of mice with identified macro-metastases in green compared to the number of mice screened with no visible metastases present in blue for each organ in both PBT and PBT-1C mice. The fractions in the middle represent the number of organs with macro-metastases over the total number of organs screened. **(d)** Donut plots displaying micro-metastases for the brain, liver, lung, pancreas, and spleen for both GEM models as described in **(c)**.

To measure metastasis, five organs relevant to clinical melanoma metastasis were then checked for the presence of TdTomato + surface metastases by imaging with a dissection microscope (Fig. 2a, b). To our surprise, we observed that the Coronin 1C-null tumors in the PBT-1C mice had more large, distant metastases (*macro-*metastases) than the control tumors in the PBT model (Fig. 2C). While PBT mice displayed no visible metastases to the brain or the liver, PBT-1C mice displayed macro-metastases in 18% and 9%, respectively. 7% of PBT mice and 5% of PBT-1C mice had macro-metastases in the lung.

Given the fact that the primary tumors grew faster in the PBT-1C mice, we suspected that metastastic tumors in distant organs in this model would also grow faster and would produce the observed trends in macro-metastases, even without differences in the rates of metastatic seeding. In order to address this question, we exploited the presence of TdTomato fluorescence in the model to develop a protocol using flow cytometry to identify micro*-*metastases that were either too small to see on the dissecting microscope or buried within the tissues (Fig. 2a). We postulated that the presence of *micro-*metastases would reflect events of metastatic seeding without the confounding factor of tumor growth. Although both the PBT and PBT-1C models showed micro-metastases, they were more prevalent in PBT-1C mice than PBT in every organ sampled with the exception of the pancreas (Fig. 2d), suggesting that in addition to primary tumor cell proliferation, metastatic seeding was also enhanced by the deletion of Coronin 1C. The most pronounced differences in the rate of micro-metastases were in the brain and liver, where 37.5% and 50% of PBT-1C mice developed micro-metastases, respectively, compared to 8.3% and 16.6% in the PBT mice. Together, these data show that deletion of Coronin 1C from the tumor cells in this model leads to significantly accelerated primary tumor growth and a trend toward enhanced metastasis.

### Coronin 1C-null cells display a higher propensity for invasion

**Figure 3:**
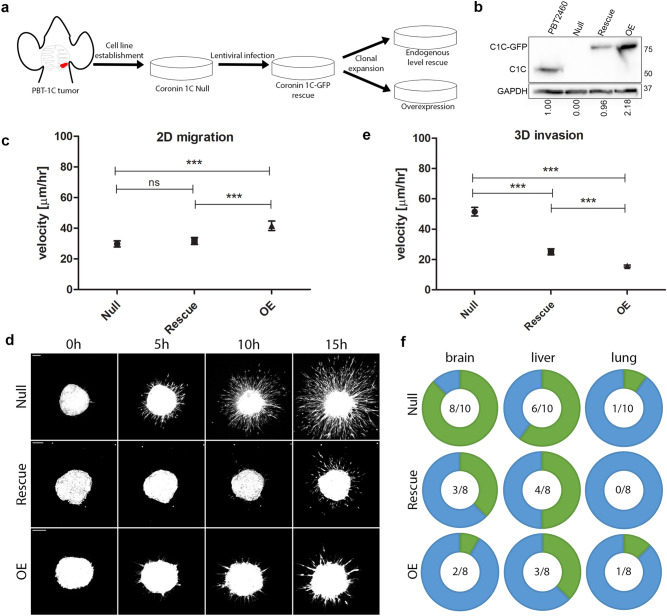
Cells lacking Coronin 1C are more invasive than their Coronin 1C-expressing counterparts. **(a)** A Coronin 1C-null cell line (*Null*) was isolated from a PBT-1C brain metastasis and infected with lentiviral Coronin 1C-GFP. This rescue line underwent clonal expansion to create one line with endogenous levels of Coronin 1C (*Rescue*) and one line with overexpressed Coronin 1C (*OE*). **(b)** Western blot of the *Null*, *Rescue,* and *OE* cell lines compared to PBT2460, a cell line isolated from a Pten/Braf melanoma tumor with endogenous Coronin 1C. Blot is cropped between C1C (Coronin 1C) and GAPDH to conserve space. An uncropped blot for each protein can be found in Suppl. Fig. 7. **(c)** Mean velocities +/− 5% CI of single *Null*, *Rescue* and *OE* cells migrating on 10 µg/mL fibronectin-coated glass. *Null* cell N = 81, *Rescue* cell N = 115, *OE* cell N = 99. Total of 3 biological replicates for each cell line. **(d)** Representative maximum intensity projection movie stills from *Null*, *Rescue*, and *OE* cell spheroids embedded in 3D collagen over 15 h after embedding. Scale bars = 100 µm. **(e)** Quantification of the mean velocities + /- 95% CI measured from individual cells invading the matrix around the main spheroid mass outlined in **(d)**. *Null* cell N = 123, *Rescue* cell N = 88, *OE* cell N = 191. 3 biological replicates for each cell line. **(f)** Donut plots displaying the number of nude mice with identified micro-metastases in green compared to those with no detectable metastasis in blue for the brain, liver, and lung dissected from nude mice injected with spheroids of *Null*, *Rescue* and *OE* cells after primary tumor ulceration. The fractions in the middle represent the number of organs with micro-metastases over the total number of organs screened. *** = P < 0.001.

To better understand the increased trend towards metastasis observed upon loss of Coronin 1C, we isolated a Coronin 1C-null cell line (referred to as Null throughout) from a macroscopic brain lesion in a PBT-1C mouse. The cells were then stably rescued with Coronin1C-GFP and single cell clones were isolated and expanded (Fig. 3a, Suppl. Fig. 2). The clone termed *Rescue*, showed Coronin 1C expression levels similar to those seen in a melanoma cell line derived previously from the Pten/Braf model^48^ containing endogenous Coronin 1C (*PBT2460*). Another clone termed *OE* (overexpression) had expression of Coronin 1C > 2 × higher than the endogenous level (Fig. 3b).

We first characterized proliferation rates of these 3 cell lines and found that *Null* cells proliferated more slowly than their Coronin 1C-expressing counterparts in 2D culture (Suppl. Fig. 2). While this observation is in line with Coronin 1C knockdown in other cancer types^34,35,49^, it also indicates that proliferation in vitro does not always accurately predict proliferation in the 3D tumor microenvironment. To compare cell motility rates, we used single cell tracking and found that *OE* cells, moved significantly faster than the *Rescue* and *Null* lines on FN-coated glass (Fig. 3c). While this is consistent with previous 2D work involving Coronin 1C in other tumor cell lines^34,35,50^, it also suggests that this assay is a poor predictor of in vivo metastasis. To better mimic the endogenous environment of the tumor, multicellular spheroids were generated from each of the three cell lines and embedded into a 3D collagen matrix. Invasion of the cells into the surrounding gel was observed over 15 h (Fig. 3d, Suppl. Vid. 1–3). *Null* cells migrated significantly faster than *Rescues*, and *OEs* moved the slowest (Fig. 3e), suggesting that this form of motility more faithfully represents with the metastasis phenotype observed in vivo.

To ensure that our in vitro results accurately reflect the in vivo phenotypes observed in the original GEM mice, spheroids generated from these cells were injected into the ears of nude mice^51^. This is a critical experiment that controls for any differences in GEM strain backgrounds that may have contributed to changes in metastatic potential. Upon primary tumor ulceration, organs were subjected to the same metastasis identification protocol that was used on the GEM mice (Fig. 2a). Tumors arising from *Null* cell spheroid injection resulted in brain and liver micro-metastases in 80% and 60% of cases, respectively, whereas the occurrence of micro-metastases fell to 25% for these organs in *OE* cells, with the *Rescue* cells displaying in an intermediate phenotype. This demonstrated a Coronin 1C-dependent decrease in metastasis (Fig. 3f) that confirms our in vivo metastasis observations in the GEM models. Minimal change in lung micro-metastases was observed between the three injected cell lines, and there were no macro-metastases observed in any of the nude mice injected with any of the cell spheroids, possibly due to faster primary tumor growth rates in this immunodeficient background that resulted in faster ulceration and therefore less time for the distant tumors to grow. These data, in conjunction with GEM model metastases and 3D cell invasion, demonstrate that the loss of Coronin 1C enhances the invasive capacity and metastasis of melanoma tumor cells to the brain and liver. It was unexpected to see such low numbers of micro-metastases to the lung considering such increase in the brain and the liver. These data, along with minimal change in lung metastases in the GEM model upon Coro1C deletion, suggests that Coronin 1C expression impacts the ability for the dissemination of metastases to the brain and liver, but not to the lung.

### Coronin 1C-null cells exhibit increased matrix degradation

**Figure 4:**
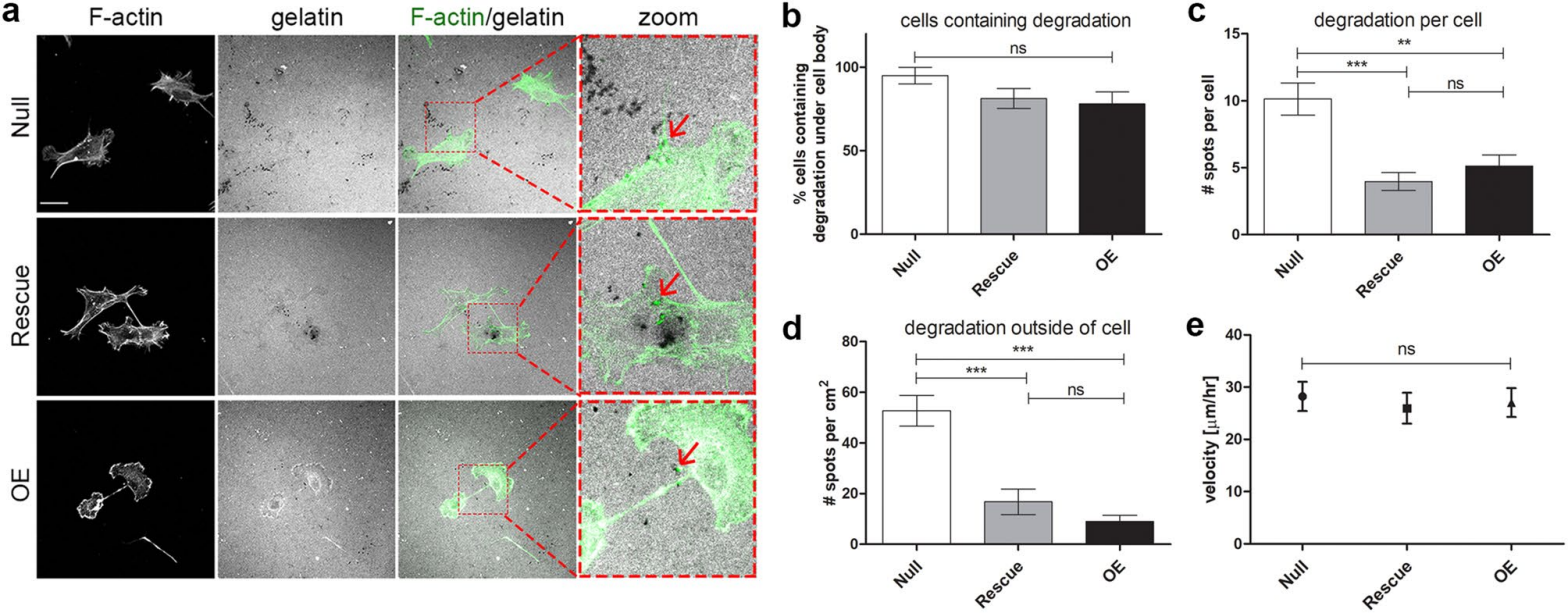
Coronin 1C null cells degrade gelatin more efficiently than Coronin 1C-expressing cells. **(a)** *Null*, *Rescue* and *OE* cells were plated on FITC-conjugated 2D gelatin matrices for 24 h, fixed, and stained for F-actin with phalloidin-568. Black spots in the gel matrix are indicative of degradation, and degradative spots of invadopodia that colocalize with actin puncta are shown with red arrows. Scale bars = 25 µm. Zoom = 3.5X. **(b)** Percent of cells that contain degraded spots of the matrix beneath the cell body. **(c)** Number of degraded spots per cell showing degradation. **(d)** Number of degraded spots per area of 2D matrix uninhabited by a cell body. N cells B-C > 30 from 3 biological replicates. N of images analyzed for D = 30 from 3 biological replicates. **(e)** Mean velocities ± 95% CI of *Null*, *Rescue* and *OE* cells migrating on 2D gelatin matrices over 16 h. N cells > 40 from 3 biological replicates. **(f)** *Null, Rescue,* and *OE* cells were plated on FN-coated glass coverslips, fixed, and surface labeled with MT1-MMP. Integrated fluorescence density was calculated for individual cells and graphed as mean + /− SEM. N > 70 for each cell line over 3 biological replicates. ** P < 0.01, *** = P < 0.001.

Since Coronin 1C has a role in suppressing migration/invasion in 3D collagen gels but not in 2D, we hypothesized that the cells lacking Coronin 1C may be able to degrade the matrix more efficiently, a crucial component of 3D migration^22,52^. To test this hypothesis, we plated cells on 2D FITC-labeled collagen matrix for 24 h to identify sites of degradation by the absence of fluorescent signal. All three cell lines formed invadopodia as defined by spots of degradation under the cells where a subset colocalized with spots of filamentous actin (F-actin)^53^. While we saw no significant change in the fraction of cells containing invadopodia with or without Coronin 1C (Fig. 4b), the *Null*s contained roughly twice as many spots of degradation under the cell body as the Coronin 1C-rescued lines (Fig. 4c).

Although Coronin 1C-null cells had more invadopodia under the cell body than the *Rescue* and *OE* cells, the most striking difference was the number of degradation spots distant from the cells (Fig. 4A, D). There were over three times more spots of degradation outside of the cell border in the *Null* cells compared to the *Rescue* and *OE* cells (Fig. 4d). One possible explanation for this result would be that Coronin 1C-null cells visit more areas of the gel during the experiment through migration, degrading the matrix via invadopodia as they moved along. However, the velocity at which the *Null* cells moved over the gelatin invadopodia substrate was not significantly altered compared to the two rescue lines (Fig. 4e).

**Figure 5:**
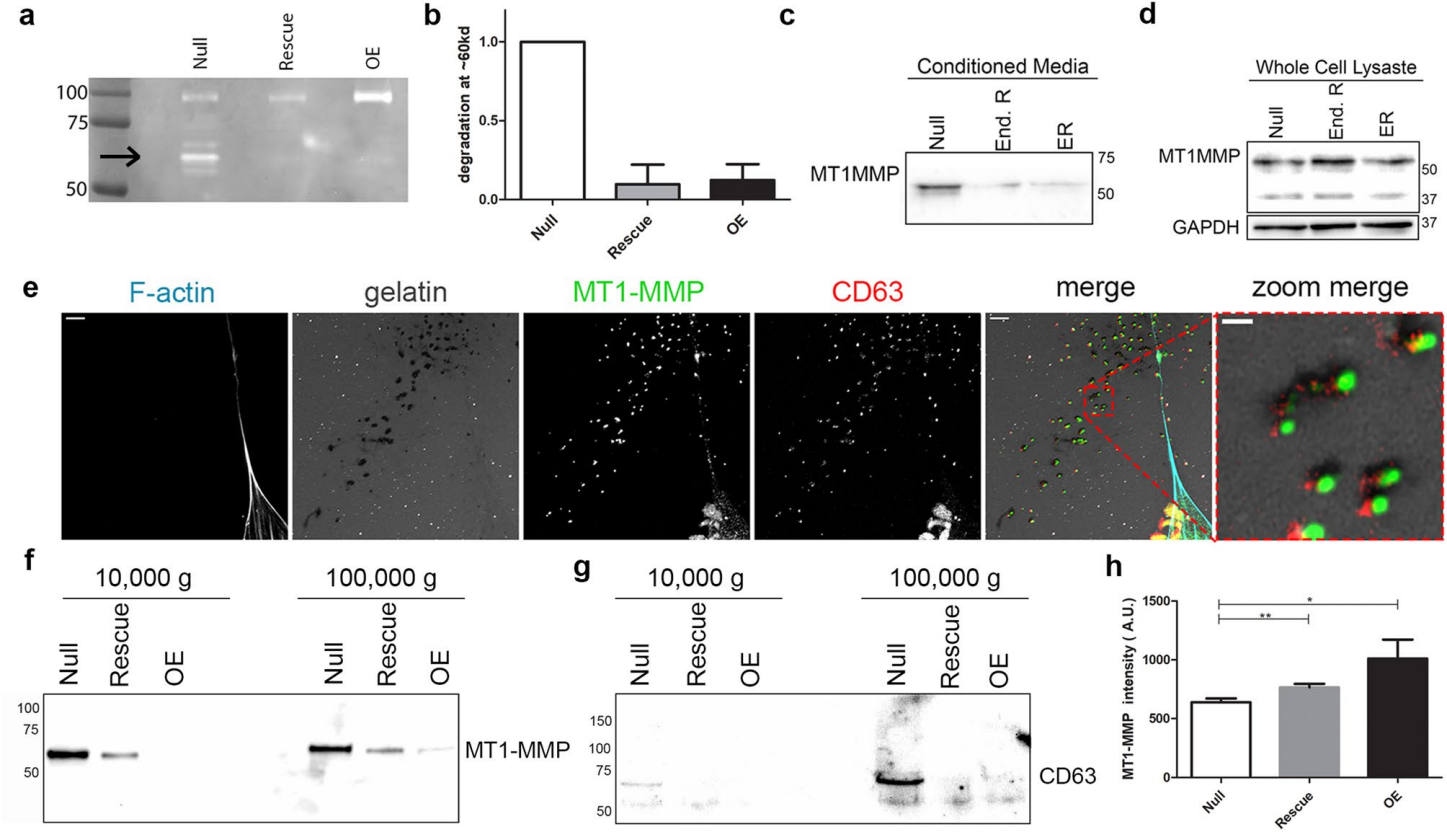
Enhanced MT1-MMP secretion coincides with matrix degradation. **(a)** Zymography gel of conditioned media from *Null*, *Rescue* and *OE* cells. Black arrow highlights band in the *Null* lane at ~ 60kd. Loading controlled by cell count at time of conditioned media collection. **(b)** Quantification of the band outlined in **(a)** across 3 biological replicates. *Rescue* and *OE* intensity was normalized to the *Null* intensity for each replicate. **(c)** Western blot of the *Null, Rescue,* and *OE* conditioned media probed with MT1-MMP. **(d)** Western blot of *Null, Rescue,* and *OE* whole cell lysates probed with MT1-MMP and GAPDH loading control in separate lanes. Blot it cropped to conserve space, uncropped blots can be found in Suppl. Fig. 7. **(e)** Immunofluorescent images of TdTomato CRISPR knockout *Null* cells plated for 24 h on 2D FITC-gelatin stained for F-actin with phalloidin-568 and immunolabeled with MT1-MMP and CD63. Full-sized image scale bars = 5 µm. Zoom image scale bars = 1 µm. **(f, g)** Western blots of EVs pelleted from conditioned media at 10,000 × *g* and 100,000 × *g* probed for MT1-MMP **(f)** and CD63 **(g)**. **(h)** Mean + /− SEM integrated fluorescent density of PM-bound MT1-MMP after staining with MT1-MMP conjugated to AlexaFluor647 without cell permeabilization. N of cells > 75 per condition across 3 biological replicates.

We therefore postulated that not all of these degradation events observed outside of the cell boundary of the *Null* cells originated from PM-localized invadopodia, but rather from a diffusible factor secreted into the extracellular space. To test this hypothesis, we harvested conditioned media from each cell line and ran a zymography assay to visualize changes in secreted degradative proteins (Fig. 5a). We identified a band of gelatin degradation at ~ 60 kD that was nearly ten times stronger in the *Null* cell conditioned media compared to the *Rescues* and *OEs* (Fig. 5b), demonstrating that the loss of Coronin 1C results in an increase in the secretion of degradative factors.

### Extracellular vesicles containing MT1-MMP, Trp1 and Rab27a are responsible for extracellular matrix degradation

We sought to identify the protein in the conditioned media responsible for the 60 kD band of degradation. MT1-MMP was a strong candidate due to its molecular weight, relevance in cancer invasion, and its unique signature in melanoma^54–57^. Western blot analysis revealed higher MT1-MMP expression in the conditioned media from *Nulls* relative to *Rescues* and *OEs* (Fig. 5c), while MT1-MMP in the cellular lysates were unchanged (Fig. 5d). To confirm that MT1-MMP localized to distant sites of degradation, we used immunofluorescent staining and observed co-localization of MT1-MMP with degraded spots both under the cells and at distant sites (Fig. 5e). These MT1-MMP-positive sites of extracellular degradation also colocalized with extracellular vesicle (EV) markers CD63 (Fig. 5e), HSC70 (Suppl. Fig. 3), and β_1_ integrin (Suppl. Fig. 3). MT1-MMP (Fig. 5f) and each EV marker (Fig. 5g, Suppl. Fig. 3) was also detectible in western blots of pelleted EVs following differential ultracentrifugation of the conditioned media in the *Null* cells while these proteins were either decreased or undetectable in the *Rescues* and *OEs.* Interestingly, we also detected increased MMP2 secretion in the *Null* cells (Sup Fig. 3F) and saw localization to sites of degradation, though on the western blot the molecular weight of ~ 75 KD ran higher than where we saw degradation on the zymography gel, and is indicative of inactive, pro-MMP2 (Suppl. Fig. 3).

Since whole-cell MT1-MMP levels were not affected by Coronin 1C expression (Fig. 5d) but were increased on EVs being secreted by Coronin 1C null cells, we tested if MT1-MMP present on the PM was affected by Coronin 1C expression. To address this question, fixed cells were labeled for MT1-MMP presented on the PM using a fluorescent conjugated primary antibody. Integrated fluorescent density was measured for individual cells to determine the quantity of MT1-MMP present on the surface. Interestingly, we found that PM-localized MT1-MMP increases as the levels of Coronin 1C expression rises (Fig. 5h), consistent with altered trafficking of MT1-MMP with and without Coronin 1C. These results suggest that MT1-MMP secreted on EVs, as opposed to the surface-presented MT1-MMP, is driving the degradation at distant sites and subsequent invasion of these cells.

**Figure 6:**
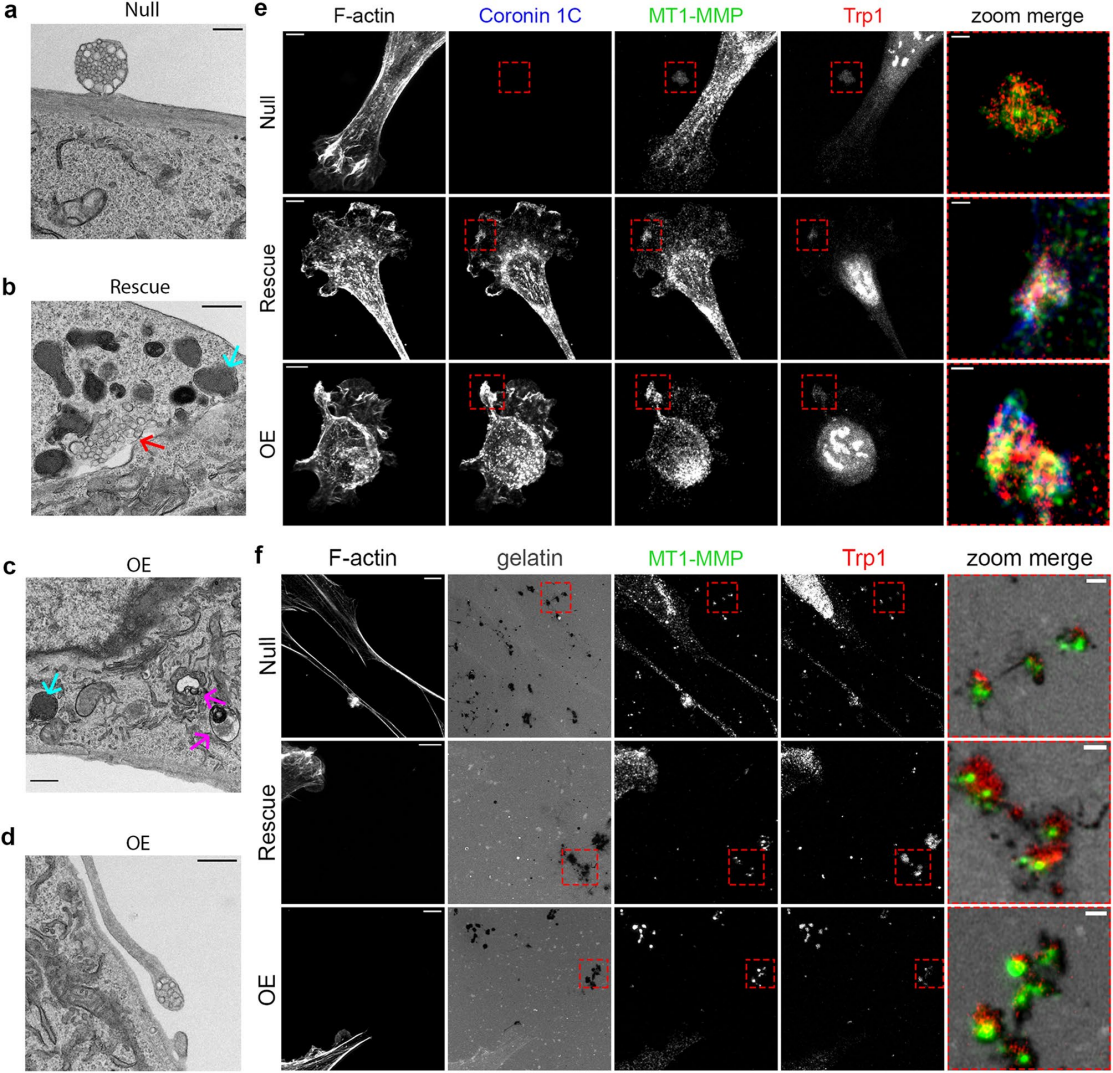
MT1-MMP EV secretion overlaps with melanosome secretion pathway. **(a–d)** TEM images of cells plated on 2D coated tissue culture dishes. **(a)** TEM of a *Null* cell secreting a multicompartmental EV. **(b)** TEM showing buildup of pigmented vesicles (blue arrows) along the PM and what looks to be an MVB not contained by a membrane within the cell body (red arrow) of a *Rescue* cell. **(c)** TEM showing a buildup of pigmented vesicles (blue arrows) and abnormal MVBs (purple arrows) along the PM of an OE cell. **(d)** TEM of a multicompartmental EV being expressed at the tip of a fingerlike projection in an *OE* cell. Scale bars for **(a)**–**(d)** = 0.5 µm. **(e)** Airyscan images of *Null, Rescue,* and *OE* cells plated on FN-coated glass, stained for F-actin with phalloidin-568 and immunolabeled with MT1-MMP and Trp1. Scale bars for full-sized images = 5 µm. Zoom image scale bars = 1 µm. **(f)** Airyscan images of *Null, Rescue,* and *OE* cells plated on FITC-labeled gelatin, stained for F-actin with phalloidin-568 and immunolabeled with MT1-MMP and Trp1. Scale bars for full-sized images = 5 µm. Zoom image scale bars = 1 µm.

Based on the evidence of altered secretion in the Coronin 1C-null cells, we sought to identify ultrastructural abnormalities indicative of changes in the secretion pathway. Transmission electron microscopy (TEM) images were taken of each of the three cell lines. The *Nulls* exhibited many multicompartmental EVs docked at the cell surface for secretion (Fig. 6a) and some separated from any surrounding cells (Suppl. Fig. 4), suggesting these structures were being released into the media. By contrast, *Rescues* and *OEs* displayed fewer EVs docked to the cell surface, and there was a noticeable increase in the amount of membrane-bound bodies built up around the PM (Fig. 6b, c, Suppl. Fig. 4). Some of these bodies bore resemblance to the EVs at the cell surface, denoted by red arrows, and others showed signs of abnormal multivesicular body development, denoted by purple arrows. An additional subset of these bodies, highlighted by blue arrows, bore a strong resemblance to granular melanosomes^58^. In cases where we captured EVs being secreted from *OE* cells, they were presented at the end of fingerlike projections (Fig. 6d, Suppl. Fig. 4) which strongly resemble a proposed mechanism of melanosome transfer where multiple melanosomes are presented at the tips of dendritic fingers for transfer to neighboring keratinocyte^59–61^.

These observations show differences in vesicular trafficking and EV secretion depending on Coronin 1C expression. We were especially intrigued by the observation of melanosome-like structures accumulating near the membrane in the *Rescue* and *OE* cells. Immunostaining with late-stage melanosome marker Trp1 revealed pockets of strong signal near the PM which colocalized with both MT1-MMP and Coronin 1C in the *Rescue* and *OE* cells (Fig. 6e). These pockets of increased PM signal were absent in *Null* cells, but Trp1- and MT1-MMP-postive spots on the glass surface near the cells were observed. On FITC-labeled gelatin, Trp1 co-localized with sites of degradation along with MT1-MMP in each of the three cell lines (Fig. 6f).

To further confirm that this phenomenon is tied to the melanosome secretion pathway, we tested the role of Rab27a, a protein critical for the trafficking and docking of melanosomes^62,63^, in MT1-MMP secretion. We found that upon Rab27a inhibition with the drug Nexinhib20 which inhibits the interaction with Rab27a and Slp1/JFC1^64^, extracellular degradation was drastically reduced (Suppl. Fig. 5). Interestingly, Rab27a also localizes to spots of extracellular degradation and was detected in the EVs pelleted from *Null* cell CM via western blotting. These data are consistent with the notion that MT1-MMP secretion is at least partially utilizing the melanosome secretion pathway, and that this process is affected by the presence or absence of Coronin 1C. This melanosome secretion pathway is lineage-specific, and thus unique to melanoma compared to other cancer types. This observation may explain the unexpected increase in metastasis upon the loss of Coronin 1C in this tumor type.

### MT1-MMP is crucial for melanoma cell invasion

**Figure 7:**
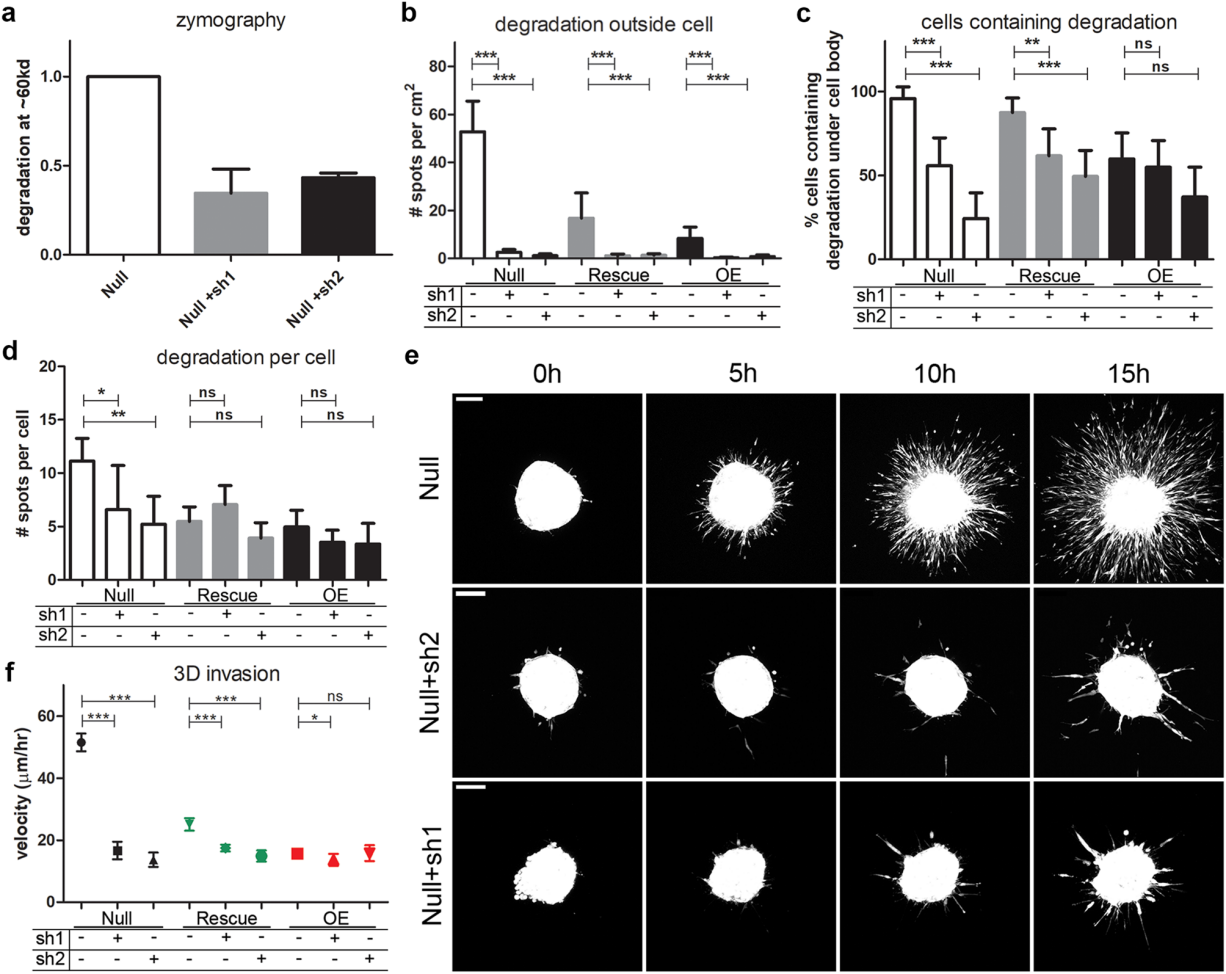
MT1-MMP is required for Coronin 1C-null cell invasive phenotype. **(a)** Mean + /− SD of intensity of degradation at ~ 60 kd from conditioned media of *Null* cells with and without treatment with two shRNA constructs targeting MT1-MMP, normalized to *Null* cells, for 3 biological replicates. **(b–d)** *Null* cells with or without treatment with 2 shRNAs targeting MT1-MMP were plated on FITC-conjugated 2D gelatin matrices for 24 h, fixed, and imaged for degradation quantification. **(b)** Number of degraded spots per area of 2D matrix uninhabited by a cell body. **(c)** Percent of cells that contain degraded spots of the matrix beneath the cell body. **(d)** Number of degraded spots per cell showing degradation. N of images analyzed for B-D = 30 from 3 biological replicates. N cells B-D > 30 from 3 biological replicates. **(e)** Representative maximum intensity projection movie stills from spheroids made from *Null* cells and *Null* cells treated with 2 shRNAs for MT1-MMP knockdown embedded in 3D collagen over 15 h after embedding. Scale bars = 100 µm. **(e)** Quantification of the mean velocities + /− 95% CI measured from individual cells invading the matrix around the main spheroid mass outlined in D. Cell N for each condition > 40 between 3 separate spheroids.

To functionally confirm that MT1-MMP was responsible for surrounding matrix degradation, we depleted MT1-MMP using two distinct shRNAs. The resulting knockdown (Suppl. Fig. 6) correlated with decreased degradation in the zymography assay of *Null* cell conditioned media (Fig. 7a). Upon plating the MT1-MMP-depleted cells on 2D gelatin, we observed a significant reduction in distant spots of degradation in *Rescue* and *OE* cells (Fig. 7b). Strikingly, we observed > 10 fold reduction in the number of degradation spots distant from the cells in the *Nulls* with MT1-MMP depletion, and these cells also showed a decrease in the percentage of cells containing degradation spots (Fig. 7c) as well as a slight decrease in the total number of degradation spots under each cell (Fig. 7d). Interestingly, MT1-MMP depletion led to only modest changes in the number of invadopodia of *Rescues* and *OE* cells suggesting that a baseline level of MT1-MMP-independent invadopodia function exists in these cells that must rely on other MMPs.

Upon MT1-MMP depletion we also observed a decreased capacity for *Null* spheroids to invade in 3D collagen (Fig. 7e, Suppl. Vids. 4–5). The velocities of the *Nulls* with MT1-MMP depletion was reduced to near the levels of the *OEs* (Fig. 7f). MT1-MMP depletion in *Rescue* cells showed slight but significant decrease in 3D cell velocity and no significant difference was observed in the MT1-MMP-depleted *OE* cells. Together, these results show that MT1-MMP secretion is critical for melanoma cells to degrade ECM and efficiently invade the surrounding matrix. Moreover, these data show that the increased invasion observed in Coronin 1C-null melanoma cells is almost entirely dependent on MT1-MMP.

## Discussion

In this study, we functionally tested the role of Coronin 1C in primary tumor growth and metastasis using a Braf/Pten Cre-inducible GEM model for melanoma^41^. Surprisingly, we found that primary tumor growth was accelerated without Coronin 1C (Fig. 1) and that both macroscopic and microscopic metastatic events were more frequent (Fig. 2, 3f). Cells isolated from these Coronin 1C-null tumors displayed a greater propensity for invasion in 3D collagen matrices compared to cells re-expressing Coronin 1C, consistent with our in vivo results (Fig. 3, Suppl. Vids. 1–3). The Coronin 1C-null cells showed a striking increase in secretion of MT1-MMP compared to rescue lines (Fig. 5), which is crucial for ECM degradation at sites distant from the cells in 2D and for 3D invasion (Fig. 7). From these observations, we conclude that the loss of Coronin 1C in melanoma leads to increased release of MT1-MMP-containing extracellular vesicles, resulting in an increased propensity for local tumor cell invasion and, more importantly, for the establishment of distant metastases. Because the MT1-MMP-containing EVs colocalize with both exosome markers as well as melanosome marker Trp1 (Figs. 5, 6, Suppl. Fig.s 3, 5), we hypothesize that secretion of these EVs reflects an aberrant, Coronin 1C-regulated melanosome secretion pathway, and that this mechanism is likely responsible for the unique role of Coronin 1C in melanoma invasion.

MT1-MMP’s role in invasion is well documented and is one of the most important MMPs for cancer progression^12–15^. The degradative protease activity of MT1-MMP is typically thought to function at the PM, with a strong enrichment at invadopodia structures^4,65^. These finger-like protrusions are crucial for tumor cell invasion in vitro and are believed to help tumor cells breach the basement membrane and other ECM barriers during several stages of metastasis in vivo^4,66–68^. In addition to the pericellular function of MT1-MMP at the PM, several studies have also reported that it is secreted into the extracellular space on EVs. MT1-MMP can be secreted both on exosomes^24,26^ as well as on microvesicles^19^ in melanoma cells. In both cases, MT1-MMP maintains the correct typology to degrade substrates in the extracellular space. However, the mechanism by which MT1-MMP is secreted on EVs and how this process is regulated remains poorly understood.

In our studies, we found extracellular spots of matrix degradation that colocalized with MT1-MMP and contained exosome markers CD63, HSC70 and β_1_ integrin (Fig. 5). While these observations suggest that these MT1-MMP-containing EVs are being released as exosomes through an MVB-mediated secretion pathway^69–72^, in actuality the story is more complicated as these structures also contain the melanosome markers Trp1 (Fig. 6f) and Rab27a (Suppl. Fig. 5). These data, along with the presence of aberrant MVBs and multivesicular EVs in our *Rescue* and *OE* cells (Fig. 6a–d, Suppl. Fig. 4), suggest that secretion of MT1-MMP-containing EVs are regulated by some kind of functional crosstalk between the MVB and melanosome trafficking machinery.

Melanoma is a uniquely aggressive cancer that has a high propensity for invasion and establishment of distant metastases. This behavior is thought to reflect the developmental history of melanoblasts/melanocytes. Consistent with this idea, melanoma cells display early-development gene signatures during tumor progression^8,73–75^ and studies illustrate how melanoma cells revert back to gene expression signatures of precursor Neural Crest Cells (NCCs) that give them growth and migratory advantages to promote tumor development^8,76^. Interestingly, MT1-MMP is highly expressed in NCCs^77^ and has recently been identified as a necessary contributor to NCC migration^78^ and these elevated levels of MT1-MMP are inappropriately re-expressed in melanoma during disease progression^12^.

Vesicular and membrane trafficking are also uniquely regulated in melanocytes and have been observed to be differentially modulated in melanoma cells compared to other cancers^7,79^. One of the core functions of melanocytes relies on a unique vesicular trafficking processes: the production, trafficking, and secretion of melanosomes, aiding in skin pigmentation and protection against damaging solar radiation. Our data demonstrating that the melanosome markers Trp1 and Rab27a colocalizes with MT1-MMP-containing EVs and subsequent matrix degradation suggests that these cells appear to be utilizing this melanocyte-specific pathway to promote invasion and metastasis, and that this process is regulated by Coronin 1C. In normal melanocytes, melanosomes mature as they are trafficked from the nucleus through the cytoplasm to the cell periphery along microtubules with the aid of Rab27a and associated motor protein myosin Va^80^. Once melanosomes reach the cell periphery, our understanding of the mechanism of their secretion and transfer becomes less clear.

Proposed mechanisms for melanosome transport include both vesicle fusion events as well as direct PM budding^80–82^. One well supported mechanism involves secretion through exocytosis of the melanin core through fusion of the melanosome with the PM^83–85^ which is consistent with both our staining data and our TEM images of intracellular and extracellular vesicles (Fig. 6, Suppl. Fig. 4). Another interesting proposal is that melanosomes are transferred to neighboring keratinocytes through the projection of filopodia-like structures with bundles of melanosomes presented at the tip for transfer to neighboring cells^59–61^, which shows a striking resemblance to the TEM images acquired of our *OE* cell line (Fig. 6d, Suppl. Fig. 4). Given the evidence that cancers can rewire lineage-specific signatures to create a growth or metastasis advantage, these trafficking pathways may be vital in understanding how Coronin 1C influences MT1-MMP secretion in our model, and why the role for Coronin 1C in melanoma seems so different from other cancer types.

Based on these expression data showing Coronin 1C overexpression/upregulation in many aggressive cancers^36–39,86^, we expected that deletion of Coronin 1C would yield a less aggressive tumor with fewer metastases. However, we found that tumors lacking Coronin 1C grew faster and were much more metastatic. Although our data are seemingly at odds with these over-expression observations, we would point out that correlation does not automatically mean causation and hypotheses derived from expression data must be tested with functional experiments. Indeed, some experiments using either RNAi depletion or over-expression in cell lines show that Coronin 1C is a positive regulator of proliferation and invasion in breast cancer and lung-squamous cell carcinoma^34,35,87^. Additionally, Coronin 1C was shown to enhance MT1-MMP trafficking to the cell surface in an orthotopic injection model of breast cancer^87^, which is again seemingly at odds with our results. However, recent data using genetic deletion of Coronin 1C in a GEM model of glioblastoma showed no change in time of survival with or without Coronin 1C^88^, further supporting the idea that Coronin 1C impacts tumor progression differently between cancer types. We postulate that the unique metastatic nature of melanoma and the underlying biology of melanocytes may help explain the context-dependent nature of Coronin 1C function in disease progression and metastasis.

Based on our data, the loss of Coronin 1C results in increased secretion of MT1-MMP-containing extracellular vesicles. Although Coronins are best known to regulate actin dynamics in lamellipodia, there is a growing body of literature supporting their function in membrane and vesicle trafficking. In HeLa cells, Coronin 1C was identified as a crucial regulator of endosomal fission from the endoplasmic^31^ and has been reported to bind directly to GDP-Rab27a to regulate endocytosis^32^ and insulin exocytosis^89^ in secretory pancreatic β-cells. In head and neck squamous cell carcinoma cells, Coronin 1B, a coronin with significant homology to Coronin 1C, has been shown to regulate exosome secretion via destabilization of the actin network at MVB docking sites through interactions with cortactin and Rab27a^71^. It is possible that Coronin 1C’s influence on the actin cytoskeleton at the PM contributes to the increased exosome secretion in our cells either through interactions with Rab27a, cortactin, and/or F-actin itself. Coronin 1C has even been shown to bind to MT1-MMP directly in glioblastoma cells^88^, but the function of this interaction has not been defined. Future studies will be directed at dissecting the precise mechanisms involved in this process.

## Materials and methods

### Reagents and materials

Antibodies for Western blotting, immunofluorescence, and IHC were purchased from Abcam (MMP14 [MT1-MMP, [EP1264Y] and CD31 [ab28364]), Takara Bio (GFP [JL8]), Thermo Fisher (GAPDH [6C5] and MMP2 [PA5-85197]), and Santa Cruz (HSC70 [B6], CD63 [MX-49.129.5], TRP1 [TA99], Rab27a [E12A-1], Trp1 [TA99], MT1-MMP [C9, AF647], and integrin β1 [A-4]). The Coronin 1C antibody was purified as described in Chan et al. 2012. 4-Hydroxytamoxifen, Nexinhib20, and 60 well Nunc MicroWell MiniTrays were purchased from Sigma Aldrich. 28 mL Nalgene Oak Ridge High-Speed PPCO Centrifuge Tubes, and 20 mm Nalgene Sealing Caps for Oak Ridge Centrifuge Tubes were purchased from Thermo Fisher. 10 KDa Amicon Ultra-15 Centrifugal Filter Units were purchased from EMD Millipore. Untreated µ-Slide 8 Well dishes were purchased from Ibidi. 35 mm Dish, No. 1.5 Coverslip, 20 mm Glass Diameter, Uncoated dishes were purchased from MatTek. A 10 µL Microsyringe model 701 and 27GA needle attachments were purchased from Hamilton.

### Genetically engineered mouse models

The PBT model used was described previously^41^. Briefly, CreER is expressed under the melanocyte-specific promotor Tyrosinase and its activity is regulated spatially and temporally by addition of 4-Hydroxytamoxifen (4-HT). Endogenous PTEN is flanked by LoxP sites on both alleles, and a WT *Braf* cDNA is flanked by LoxP sites and followed by the V600E mutant form expressed from the endogenous promoter upon deletion of the WT cDNA. A Lox/stop/Lox site precedes a TdTomato gene, which upon activation with Cre is deleted allowing for TdTomato expression. The PTB-1C model was derived from the PBT model with homozygous floxed *Coro1C* alleles bred in^44^. Breeding, care, and maintenance of each strain were carried out in accordance to UNC’s Division of Laboratory Animal Medicine- and Institutional Animal Care and Use Committee-approved protocols and standards. Genotyping for *correct PTEN, BRAF, TdTomato, Cre*, and *Coro1C* is completed from DNA isolated from toe clips of pups before use in experimentation.

### Tumor induction and measurement in GEM models

Tumors were induced in PBT and PBT-1C mice by local application of 4-HT to the left ear of 7–8-week-old PBT and PBT-1C mice of both sexes as described previously^41^. Briefly, 1 µL of 20 mM 4-HT solution in DMSO, 100% ethanol, and orange-6 dye was applied under anesthesia for precisely nine minutes before the area was thoroughly washed with 70% ethanol. Tumor growth was quantified by measuring the thickness of the ear where the tumor was induced every seven days by caliper while the mice were anesthetized with 1.5% isoflurane.

### Cell lines

All cells were cultured in DMEM with 10% FBS at 37 °C and 5% CO_2_. A Coronin 1C-null cell line (referred to all *Null* throughout the paper) was isolated from a brain metastasis from a male PBT-1C mouse induced with 4-HT by surgical resection of the tumor, dissociation in 0.25% trypsin/EDTA, and selected by FACS for TdTomato expression. Western blot analysis confirmed that the cell line was null for Coronin 1C as well as Pten. Mouse Coronin 1C fused to GFP on its C-terminus in the lentiviral vector pLL5.0^90^ was packaged into viral particles using HEK293FT cells. Null cells were incubated with this virus for 36 h. Infected cells were selected by FACS sorting for GFP expression and expanded. Single GFP-positive cells were sorted into a 96-well plate for clonal expansion by the UNC Flow Cytometry Core Facility. Clonal populations were screened for Coronin 1C expression levels using Western blotting. One clonal population was identified as having endogenous Coronin 1C expression compared to line PBT2460 isolated from the PBT model^42^ and we designated this line *Rescue*. Another clonal population showed ~ 2 × higher Coronin 1C expression and was called *OE*. TdTomato-negative cell lines were generated by targeting TdTomato using the Zhang Lab lentiviral CRISPR toolbox protocol and lentiCRISPR v2 construct^91,92^. Cells infected with the construct were selected using 10 µM puromycin, and the cells were then sorted for lack of TdTomato expression using FACS.

### Macro-metastasis identification

Mice were sacrificed when the primary tumor began to ulcerate via CO_2_ euthanasia and cervical dislocation according to UNC’s Institutional Animal Care and Use Committee guidelines. Organs were harvested, placed in sterile PBS and imaged with an Olympus MVX10 macroscope system. TdTomato + macro-metastases were identified using fluorescence and appropriate filter sets in conjunction with Metamorph software.

### Micro-metastasis identification

Organs that had been scanned for macro-metastases were prepped for micro-metastasis identification protocol adapted from a previous method^93^, then transferred to 6 cm dishes and minced with a razor blade in 0.5 mL of sterile PBS. The dish was then rinsed with 1 mL of PBS and all the contents transferred to a 15 mL conical tube. These were spun at 1,000 rpm for three minutes and the supernatant was removed. The pellets were then resuspended in 0.25–1 mL of 300 U/mL collagenase and 100 U/mL hyaluronidase in DMEM, incubated for 1 h at 37 °C with vortex mixing at 30 min. The mixtures were again pelleted and the supernatant was removed, followed by wash with 1 mL PBS. At this point, brain tissue was set aside because it did not require further dissociation. The remaining organ mixtures were resuspended in 0.25% trypsin/EDTA and incubated for 21 min at 37 °C with a vortex mix every 7 min, before being spun down and rinsed with 1 mL PBS. All dissociated organs were resuspended in 0.5 mL ice cold PBS and fixed with 4.5 mL ice cold 4% PFA for 15 min. Cells were then spun down and rinsed with 1 mL of PBS for 5 min and stored at 4 °C for no longer than 48 h before analysis. Cells were prepped for sorting according to protocol outlined previously^94^ and profiled with a Bio-Rad S3 Cell Sorter. TdTomato + threshold was set by the profile of TdTomato-expressing Coronin 1C-defincient *Null* cell line as outlined below. Uninduced PBT and PBT-1C mice (not treated with 4-HT) were used as a negative control by profiling each organ for endogenous TdTomato expression. For each organ type, the mean number of cells that fell inside of the TdTomato + threshold plus the 95% confidence intervals were calculated so that each organ had its own unique threshold based on endogenous autofluorescence of the tissue. Any induced mouse organ TdTomato profile that fell above the control 95% confidence interval was considered a positive hit for a micro-metastasis.

### Immunohistochemistry

Tumors were harvested from the base of the ear, fixed in 4% PFA for 24 h at 4 °C and preserved in 30% sucrose at 4 °C. Fixed tumors were washed for 24 h in PBS and paraffin embedded for sectioning. Sections underwent heat-induced epitope retrieval and were chromogen stained for CD31 with Vector Red and counterstained with hematoxylin. Sectioning and staining were preformed by the Histology Research Core Facility in the Department of Cell Biology and Physiology at the University of North Carolina at Chapel Hill.

### Depletion of MT1-MMP by shRNA

shRNAs against mouse MT1-MMP were acquired from the UNC-CH Lenti-shRNA Core Facility in pLKO.1 vectors. Lentiviruses expressing these shRNAs were generated using standard packaging methods in HEK293FT cells as described previously^95^ and were used to infect *Null, Rescue,* and *OE* cells for 36 h. Infected cells were selected by 10 µM puromycin and knockdown was assessed by western blotting. All experiments were completed with three passages following puromycin selection. The targeting sequences for MT1-MMP were: (sh1) CCATCAATACTGCCTACGAAA; (sh2) GCAGTGATGAAGTCTTCACAT.

### Zymography

80% confluent 6 cm dishes were rinsed with PBS and covered with 4 mL of serum-free DMEM. After 24 h, 3.5 mL of this conditioned media was removed and concentrated to 250 µL using Amicon Ultra-15 Centrifugal Filters. Conditioned media volumes were normalized according to cell count from the dishes of origin and incubated with 2 × SDS loading buffer without 2-mercaptoethanol for 20 min at 37 °C before being loaded into a 10% SDS gel containing 0.1% porcine gelatin. Gels were run at 200 V packed on ice until the 37kd marker ran off of the gel, then washed four times for 30 min each at room temperature with wash buffer (25 mM Tris pH 7.6, 5 mM CaCl_2_, 3 mM NaN_3_, 0.25 nM ZnCl_2_, and 1.25% Triton-X100 in water). The gel was then washed with ddH_2_O for 30 min before incubation for 36 h at 37°C in incubation buffer composed of 25 mM Tris pH 7.6, 5 mM CaCl_2_, 3 mM NaN_3 _and 0.375 M NaCl in water. The gel was then quickly rinsed in distilled water before staining with 0.1% Coomassie Blue solution for 1 h. After destaining for about 30 min, the gel was imaged using a Bio-Rad ChemiDoc MP Imaging System. Band intensity was quantified using Image Lab 6.0.1 software and graphed in GraphPad Prism.

### Western blotting and immunostaining

Western blotting was performed as described previously^96^. Conditioned media loading was controlled by normalizing the volume to the number of cells counted from the dish which the media was removed. Band intensity was quantified using Image Lab 6.0.1 software and graphed in GraphPad Prism. Immunostaining of cells on either 2D gelatin matrix or fibronectin-coated glass was done following a previously published protocol^97^.

### Cell spheroid generation: hanging droplet method

Cells in culture were suspended at 100,000 cells/mL. 24 µL of suspension was added to each well of a Nunc MicroWell MiniTray and inverted. Dishes were placed inside of a 15 cm culture dish humidified with 8 mL of PBS inside an open 6 cm dish. Spheroids were left to coalesce for 4 days at 37 °C 5% CO_2_ before use.

### Nude mouse spheroid injection

Cell spheroids plus 1–1.5 µL of sterile PBS were injected into the left ear of nude mice anesthetized with 1.5% isoflurane using a Hamilton Microliter Syringe with a 27GA needle^29^. Primary tumor growth was monitored weekly and imaged on an Olympus MVX10 macroscope system. Mice were sacrificed via CO_2_ euthanasia and cervical dislocation according to UNC’s Institutional Animal Care and Use Committee guidelines when the primary tumor began to ulcerate and were processed by the macro- and micro-metastasis identification protocol.

### Microscopy

Fixed-cell and live-cell images of 3D spheroid invasion, 2D matrix degradation, and cells plated on glass coverslips were captured with a Zeiss LSM800 microscope with a 10 ×, 40 ×, or 63 × objective using both confocal and airyscan settings. Movies and images were processed on both Zen Blue and Fiji software. IHC slides were imaged using an Olympus BX51 microscope with a 40 × objective using Metamorph software. TEM was performed by the University of North Carolina’s Microscopy Services Laboratory.

### 2D collagen matrix degradation

Acid-washed coverslips were coated with a 2D FITC-conjugated collagen matrix previously described^94^. Equal numbers of each cell type were plated onto the gel-coated coverslips in 12 well dishes for 24 h before fixation and staining. Degradation spots were counted manually. Calculations of matrix area devoid of cells was determined by using a custom MATLAB script to identify cell area and subtracting that value from the total area of the image. Briefly, the code uses the image processing toolbox to threshold the cell boundary from fluorescent cell images and calculates the area within that boundary in order to determine the area of matrix in uninhabited by a cell body. The code used in this study and a sample data set is openly available on GitHub along with auxiliary scripts accessible for download at https://github.com/maxhocken/SuperfineBear.

### 3D matrix invasion

Gel matrix composed of 1 mg/mL rat tail collagen, 1 × DPBS, and 3.3 mM NaOH dissolved in sterile water. 100 µL of gel mix was used to coat the bottom of each glass-bottomed chamber slide well and solidified at 37 °C for 1 h. One spheroid was suspended in 100 µL of gel mix and pipetted on top of the primary gel layer and solidified for 1 h at 37 °C and then overlaid with 100 µL of DMEM to avoid drying out. Chamber slides were then imaged every 10–20 min for 16 h with a Zeiss LSM800 confocal microscope in 51 Z planes across 200 µm. Maximum intensity projection movies were generated with Zen Blue software and invading cells were tracked using the manual tracking tool on Fiji. Velocities were calculated using the chemotaxis plugin on Fiji. Temperature, humidity, and CO_2_ levels were maintained by a Tokai Hit chamber regulator.

### Surface MT1-MMP quantification

Cells were plated on FN-coated glass coverslips and fixed with 4% PFA. After three 10-min washes with PBS, cells were blocked for 30 min in 10% BSA and 10% NGS in PBS. Without permeabilizing the cells, surface MT1-MMP was labeled via incubation with AlexaFluor647-conjugated MT1-MMP primary antibody for 1 h in 1% BSA in PBS. The cells were rinsed three times for 10 min with PBS, then mounted onto slides for imaging using a Hamamatsu Orca-flash 4.0LT camera on a Zeiss800 microscope and 40 × objective. MT1-MMP surface expression was quantified by calculating the integrated pixel density of individual cells across 3 biological replicates, as previously described^98^. Briefly, images were imported into Fiji and the TdTomato signal from the cells was used to carefully trace an outline for each cell. Integrated pixel density of the AlexaFluor647 signal was measured and calculated for each individual cell using Fiji’s measurement tool.

### Ultracentrifugation

Three 10-cm dishes of 80% confluent cells were rinsed with PBS before incubation with 10 mL each of serum-free DMEM for 48 h. CM was collected and cell debris was spun out at 5,000 *g* for 15 min and discarded. 25 mL of supernatant was transferred to ultracentrifuge tubes and spun at 10,000 *g* for 30 min. Supernatant was transferred to clean ultracentrifuge tubes and spun at 100,000 *g* for 2 h. The pellets from the 10,000 *g* and 100,000 *g* spins were resuspended in 400 µL of PBS containing protease inhibitor cocktail and run out in western blots for protein detection.

### 2D migration assay

Equal numbers of cells were either plated on 2D collagen matrix or glass-bottomed Mat-Tek dishes coated with 10 µg/mL fibronectin for 1 h and rinsed with PBS. Cells were given 4 h to adhere before imaging every 10 min with a VivaView microscope for 16 h. Movies were generated with Metamorph software and individual cells were tracked in Fiji using the manual tracking tool. Velocities were calculated using the Fiji Chemotaxis plugin.

## Supplementary information

Supplementary file1

Supplemental Video 1

Supplemental Video 2

Supplemental Video 3

Supplemental Video 4

Supplemental Video 5

## Data Availability

The datasets generated during and/or analyzed during the current study are available from the corresponding author on reasonable request.
