# Supplementary material for: Coronin 1C inhibits melanoma metastasis through regulation of MT1-MMP-containing extracellular vesicle secretion
Source: Sci Rep. 2020 Jul 20;10:11958. doi: 10.1038/s41598-020-67465-w (PMC7371684; doi:10.1038/s41598-020-67465-w)
Supplement: Supplementary file 1 — Supplementary file1 [file 41598_2020_67465_MOESM1_ESM.pdf]

Coronin 1C inhibits melanoma metastasis through regulation of MT1-MMP-containing extracellular vesicle secretion

Alicia C. Tagliatela[1,2], Stephanie C. Hempstead[2], Priya S. Hibshman[1,2], Max A. Hockenberry[2], Hailey E. Brighton[1,2], Chad V. Pecot[2,3], James E. Bear[1,2]*

[1] Department of Cell Biology and Physiology, University of North Carolina at Chapel Hill, Chapel Hill, NC, 27599, USA

[2] Lineberger Comprehensive Cancer Center, University of North Carolina at Chapel Hill, Chapel Hill, NC, 27599, USA

[3] Division of Hematology and Oncology, University of North Carolina at Chapel Hill, Chapel Hill, NC, 27599.

Correspondence: * jbear@email.unc.edu

SUPPLEMENTAL FIGURES

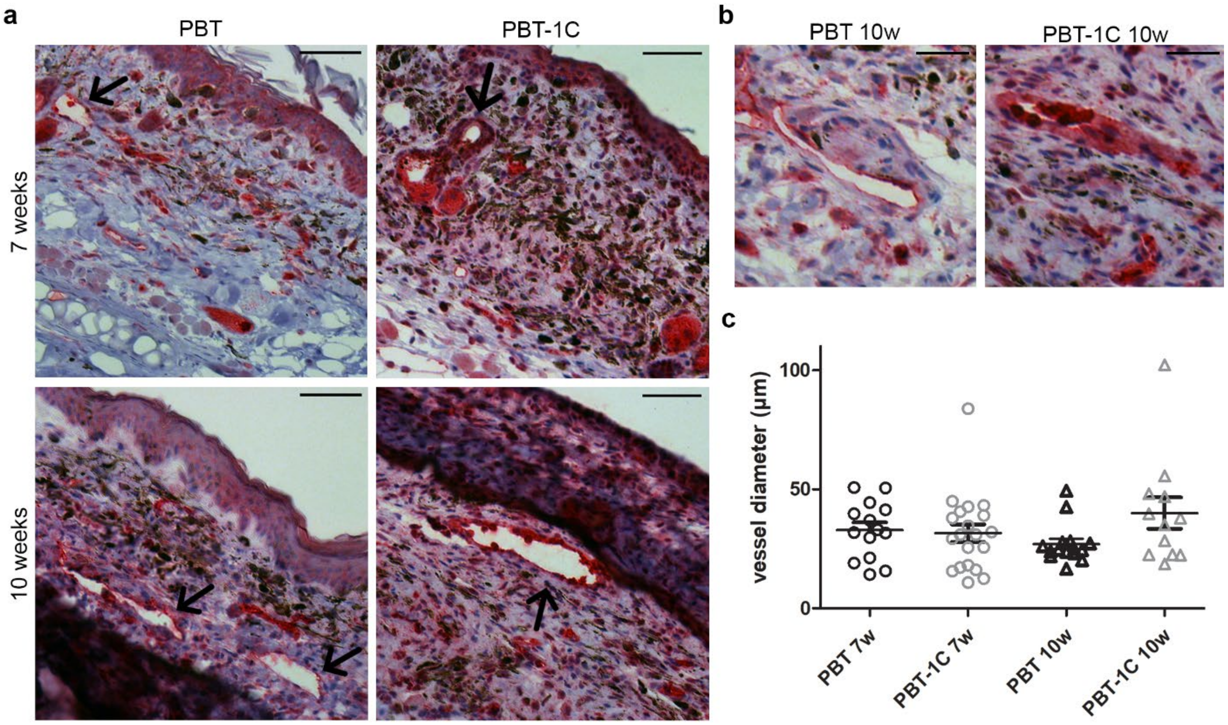

Supplemental Figure 1: IHC of primary tumors reveals greater CD31 intensity around blood vessels in PBT-1C tumors

A) Representative images of chromogenic IHC staining of CD31 with ImmPACT Vector Red and hematoxylin counterstain. Primary tumors were harvested 7 and 10 weeks after induction with 4-hydroxytamoxifen. Blood vessels with a visible lumen surrounded with CD31 signal are highlighted with black arrows. Scale bars = 50 µm. B) Representative zoomed in images of slides imaged in (A) showing the intensity of CD31 signal surrounding blood vessel lumen in PBT and PBT-1C primary tumors 10 weeks after induction with 4-hydroxytamoxifen. Scale bar = 25 µm. C) Mean +/- SEM of blood vessel diameter at widest section measured from images

taken from IHC described in (A). N of one tumor per condition, >12 vessels for each condition. Unpaired T tests revealed no significant differences between conditions.

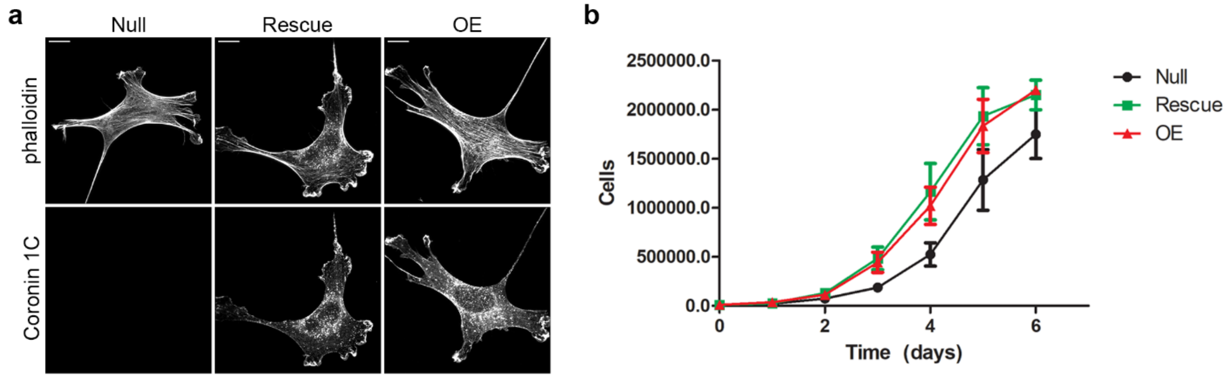

Supplemental Figure 2. Coronin 1C rescue shows predicted localization and increases proliferation rate

A) Airyscan images of fixed *Null, Rescue*, and *OE* cells with Coronin 1C-GFP expression, stained for F-actin with phalloidin-647. 63x objective, scale bar = 10 μm. B) Growth curve of *Null, Rescue,* and *OE* cells with 10,000 cells plated on day 0. Time points taken every 24 hours. Data points represent mean cell count +/- SEM over 3 biological replicates for each cell line.

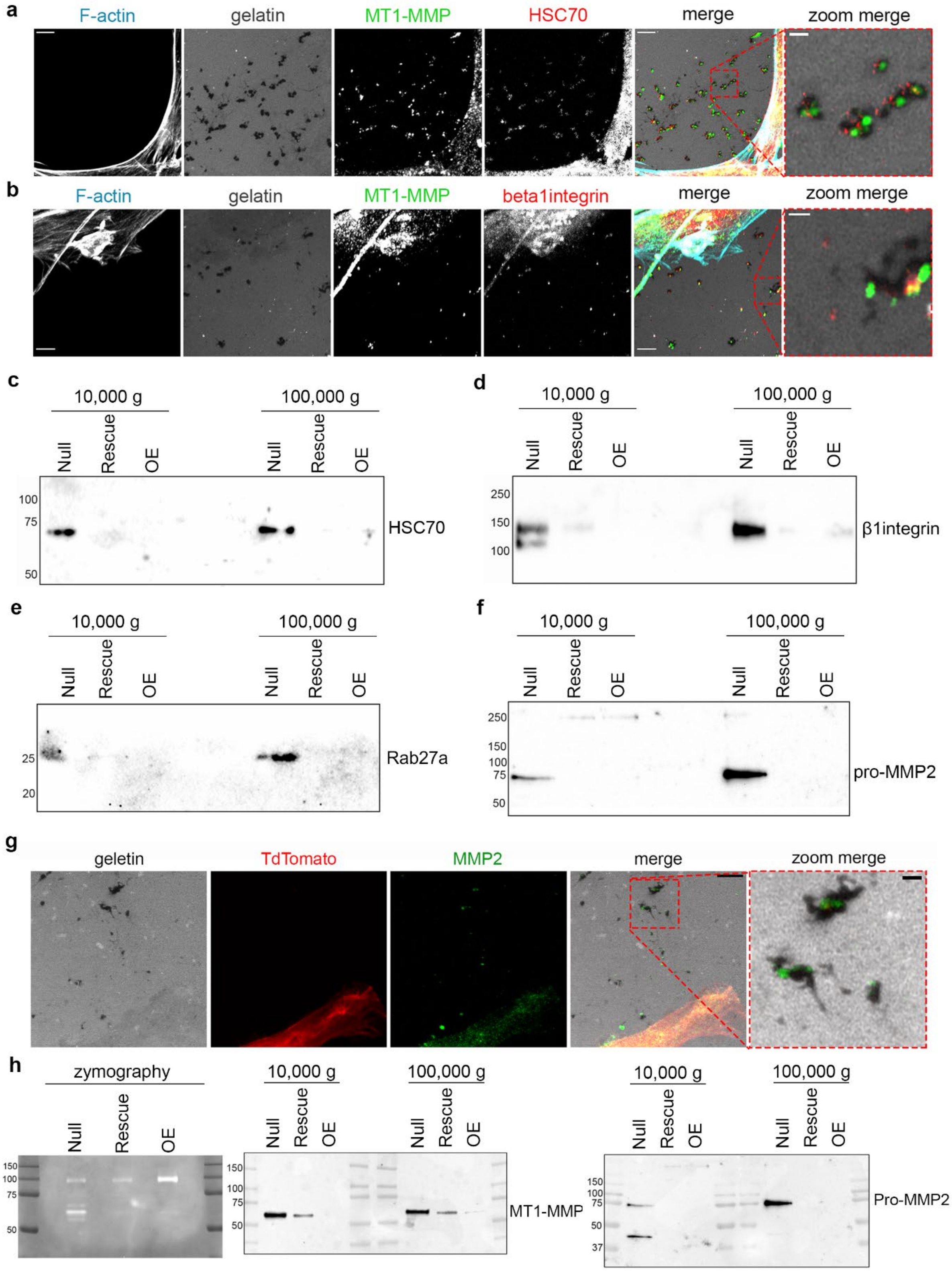

Supplemental Figure 3. MMPs and vesicle markers are identifiable by IF and western blotting of resuspended pellets following ultracentrifugation of conditioned media in *Null* cells

A-B) Immunofluorescent images of TdTomato CRISPR knockout *Null* cells plated for 24 hours on 2D FITC-gelatin stained for F-actin with phalloidin-568 and immunolabeled with HSC70 (A) and $\beta_1$integrin. Full-sized image scale bars = 5 μm. Zoom image scale bars = 1 μm. C-F) Western blots of EVs pelleted from conditioned media at 10,000 x g and 100,000 x g probed for HSC70 (C), $\beta_1$integrin (D), Rab27a (E), and MMP2 (F). G) Immunofluorescent images of *Null* cells plated for 24 hours on 2D FITC-gelatin immunolabeled with MMP2. Full-sized image scale bars = 5 μm. Zoom image scale bars = 1 μm. H) Zymography gel of conditioned media from *Null*, *Rescue* and *OE* cells side by side with western blots of EVs pelleted from conditioned media at 10,000 x g and 100,000 x g probed for MT1-MMP and MMP2 overlaid with white light images so that the transferred protein ladder is visible along with the antibody detection.

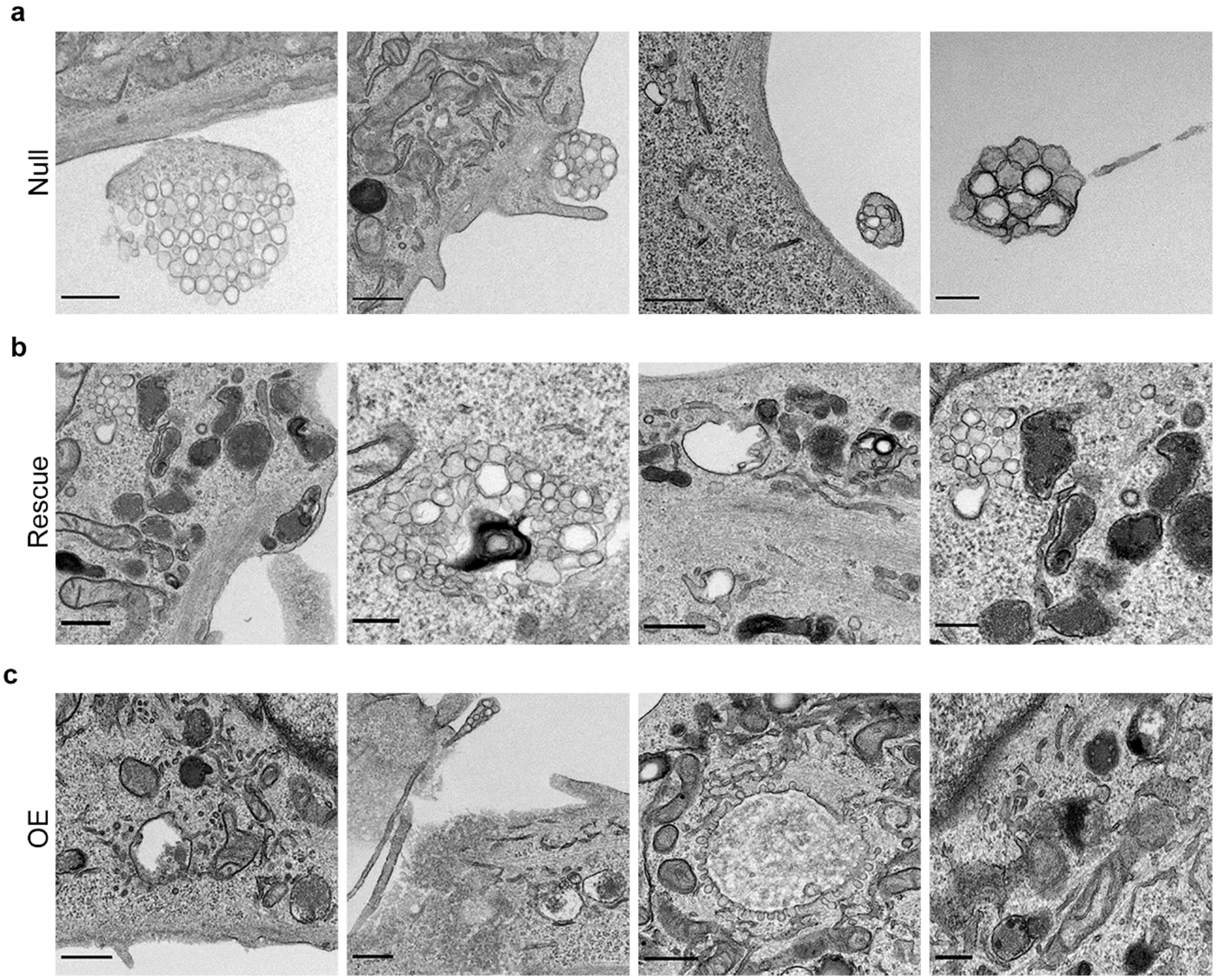

Supplemental Figure 4. Coronin 1C re-expression results in increased vesicle buildup, and abnormal vesicle production and presentation

A) Transmission electron microscopy of *Null* cells shows release of multicompartmental EVs and fewer vesicles built up in the cytoplasm along the PM. Scale bars left to right: 500 nm, 500

nm, 500 nm, 200 nm. B) Transmission electron microcopy of *Rescue* cells showing an abundance of various abnormal vesicular bodies built up in the cytoplasm. Scale bars left to right: 500 nm, 250 nm, 500 nm, 250 nm. C) Transmission electron microscopy of *OE* cells displaying an abundance of various abnormal vesicular bodies built up in the cytoplasm and presentation of vesicles at the end of filopodia-like projections. Scale bars left to right: 500 nm, 250 nm, 500 nm, 250 nm.

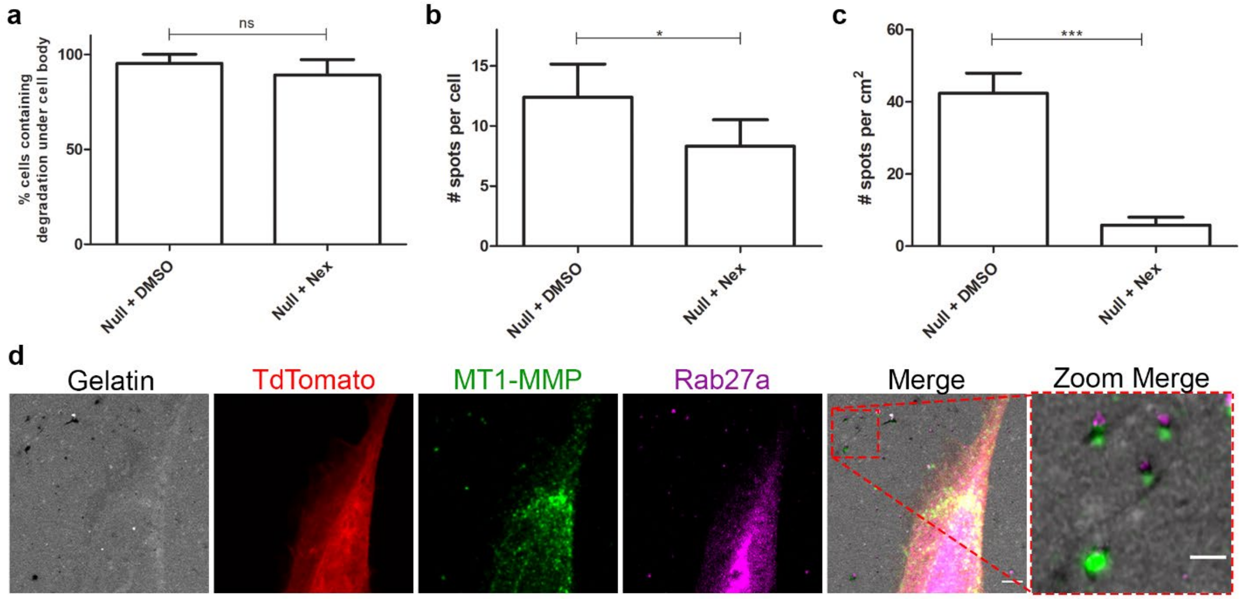

Supplemental Figure 5. Inhibition of Rab27a with Nexinhib20 reduces extracellular degradation in *Null* cells

Null, *Rescue* and *OE* cells were treated with either 1 μM Nexinhib20 or equal volume DMSO for 1 hour, then plated on FITC-conjugated 2D gelatin matrices for 24 hours in media containing either drug or DMSO. Cells were fixed and stained with MT1-MMP and Rab27a. Black spots in the gel matrix are indicative of degradation of the gelatin layer. A) Percent of cells that contain degraded spots of the matrix beneath the cell body. B) Number of degraded spots per cell showing degradation. C) Number of degraded spots per area of 2D matrix uninhabited by a cell body. N cells A-C >30 from 3 biological replicates. D) Example image of *Null* cell treated with 1 μM Nexinhib20. MT1-MMP and Rab27a localize with spots of degradation. Scale bar in merge = 2.5 μm and scale bar in zoom merge = 1 μm. * = P <.05, *** = P <0.001, ns = P >.05.

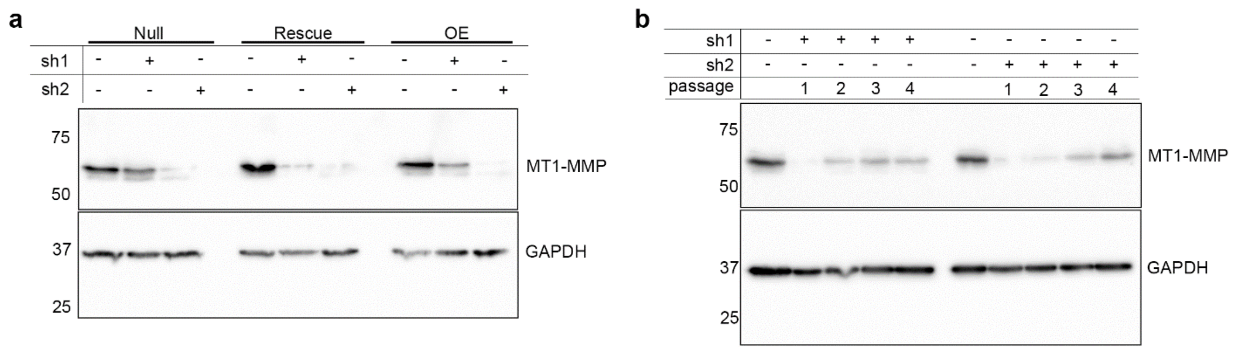

Supplemental Figure 6. Treatment with MT1-MMP shRNAs results in knockdown stable for 3 passages

A) Western blot of whole cell lysates of *Null, Rescue,* and *OE* cells 2 passages after infection with MT1-MMP-targeting shRNA lentivirus and selection via puromycin treatment. Blots were probed with MT1-MMP and GAPDH for loading control, run with the same sample and volume but on a different gel, and cut for ease of probing with antibodies at low volume. B) Western blot of whole cell lysates from *Null* cells infected with MT1-MMP-targeting shRNA lentivirus 1-4 passages after puromycin treatment, compared with *Null* cells with mock infection. Blots were probed with MT1-MMP and GAPDH for loading control, run with the same sample and volume but on a different gel, and cut for ease of probing with antibodies at low volume.

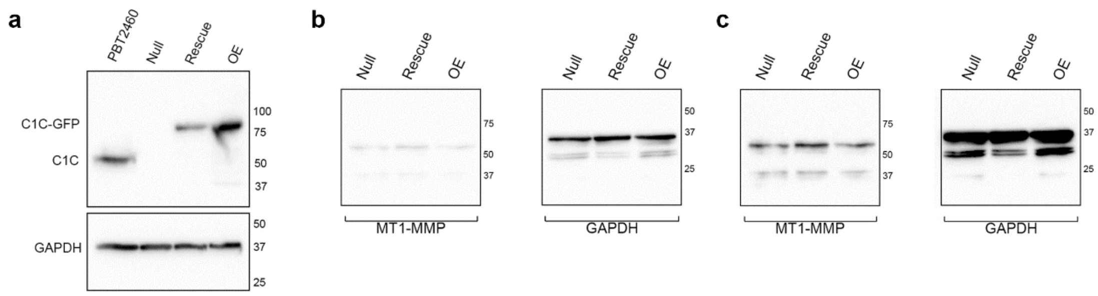

Supplemental Figure 7. Western blots of whole cell lysates to supplement main figures

A) Western blot of the *Null*, *Rescue,* and *OE* cell lines compared to PBT2460, a cell line isolated from a Pten/Braf melanoma tumor with endogenous Coronin 1C. Blots were probed with MT1-MMP and GAPDH for loading control, run with the same sample and volume but on a different gel, and cut for ease of probing with antibodies at low volume. B) Low exposure image of a western blot of *Null, Rescue,* and *OE* WCLs probed with MT1-MMP and GAPDH loading control. Blots were probed with MT1-MMP and GAPDH for loading control, run with the same sample and volume but on a different gel, and cut for ease of probing with antibodies at low volume C) High exposure image of the same western blot pictured in B.

SUPPLEMENTAL VIDEO TITLES

Supplemental Video 1. *Null* cell spheroid invasion in 3D collagen matrix

Supplemental Video 2. *Rescue* cell spheroid invasion in 3D collagen matrix

Supplemental Video 3. *OE* cell spheroid invasion in 3D collagen matrix

Supplemental Video 4. *Null*+MT1-MMP sh1 cell spheroid invasion in 3D collagen matrix

Supplemental Video 5. *Null*+MT1-MMP sh2 cell spheroid invasion in 3D collagen matrix
